# Analysis of the Fungal Diversity and Community Structure in Sichuan Dark Tea During Pile-Fermentation

**DOI:** 10.3389/fmicb.2021.706714

**Published:** 2021-08-05

**Authors:** Kuan Yan, Manzar Abbas, Lina Meng, Hongbing Cai, Zhang Peng, Quanzi Li, Ahmed H. El-Sappah, Linfeng Yan, Xianming Zhao

**Affiliations:** ^1^Faculty of Agriculture, Forestry and Food Engineering, Yibin University, Yibin, China; ^2^Key Laboratory of Sichuan Province for Refining Sichuan Tea, Yibin, China; ^3^Sichuan Province Tea Industry Group Co., Ltd., Yibin, China; ^4^State Key Laboratory of Tree Genetics and Breeding, Chinese Academy of Forestry, Beijing, China; ^5^Research Institute of Forestry, Chinese Academy of Forestry, Beijing, China; ^6^Department of Genetics, Faculty of Agriculture, Zagazig University, Zagazig, Egypt

**Keywords:** Sichuan dark tea, pile-fermentation, fungal community, high-throughput sequencing, 16S rRNA (16S rDNA)

## Abstract

The fungi present during pile-fermentation of Sichuan dark tea play a pivotal role in the development of its aroma and physical characteristics. Samples of tea leaves were collected on days 0 (YC-raw material), 8 (W1-first turn), 16 (W2-second turn), 24 (W3-third turn), and 32 (W4-out of pile) during pile-fermentation. High-throughput sequencing revealed seven phyla, 22 classes, 41 orders, 85 families, 128 genera, and 184 species of fungi. During fermentation, the fungal diversity index declined from the W1 to W3 stages and then increased exponentially at the W4 stage. A bar plot and heatmap revealed that *Aspergillus*, *Thermomyces*, *Candida*, *Debaryomyces*, *Rasamsonia*, *Rhizomucor*, and *Thermoascus* were abundant during piling, of which *Aspergillus* was the most abundant. Cluster analysis revealed that the W4 stage of fermentation is critical for fungal growth, diversity, and the community structure in Sichuan dark tea. This study revealed the role of fungi during pile-fermentation in the development of the essence and physical characteristics of Sichuan dark tea. This study comes under one of the Sustainable Development Goals of United Nations Organization (UNO) to “Establish Good Health and Well-Being.”

## Introduction

Sichuan dark tea is manufactured by processing a mixture of fresh leaves and mature branches collected from tea plants (*Camellia sinensis*; Wang et al., 2018b). Dark tea is one of the six famous tea types produced in the northwest and southwest borders of China, and the production of this tea is a significant contributor to the livelihood of ethnic minorities in China (Wang et al., 2016). Sichuan dark tea is a post-fermented tea with a unique flavor, can be stored for long-time, and can be brewed multiple times. It has demonstrated health benefits including anti-hyperglycemia, anti-hypertension, and anti-hyperlipidemia effects (Wang et al., 2016, 2018b). Dark tea is one of the richest sources of biologically active compounds including vitamins, amino acids, organic acids, polyphenols, and trace elements (Chen et al., 2017; Xu et al., 2020). It is also a widely used medicinal plant as a preventive, especially in traditional Chinese medicine (Peng et al., 2014; Liu, 2016). However, the over-use of dark tea may have some negative health effects, such as caffeine causes heartburn, nausea, dizziness, and poor sleep quality (Srinivasan et al., 1997), while tannins intercalate with iron and refrain, it being absorbed by human digestive system (Walch et al., 2012).

The pile-fermentation process of dark tea comprises the following steps: fixation, rolling, piling, and drying (Wang et al., 2016). During fixation, high temperature causes oxidase inactivation, dehydration, soften leaves, and develop aroma. In rolling, tea leaves are chopped into small strips and squares and finally milled to disrupt cell walls of tea cells. During piling, the tea leaves are piled under warm, humid conditions, during which fermentation by associated microbes occurs, called as pile-fermentation. Finally, unique dark color and aroma are developed by baking during drying process. The microorganisms involved in the pile-fermentation process of dark tea are unique among tea types. Notably, although a considerable number of microbes in raw tea is killed after fixation, many microbes retained during subsequent processing (Li et al., 2017a). Piling is a key determinant of the color, aroma, taste, and shape of dark tea. The unique flavor and essence of dark tea are predominantly developed through polyphenol oxidation, the metabolic activities of microbes, biochemical processes, such as those catalyzed by extracellular enzymes, and physicochemical properties, such as heat and humidity, during pile-fermentation (Zhang et al., 2016a; Wang et al., 2018b). For example, *Penicillium* can hydrolyze the fiber in the tea during piling, increase its sugar content, and contribute to the taste of dark tea. *Aspergillus niger* and yeasts produce a variety of hydrolytic enzymes that participate in mutual transformation reactions among the various substances in tea. In piling, the raw tea materials undergo enzymatic reactions and fermentation, which are driven by a series of microorganisms to develop the characteristics of quality dark tea, that is, a dark leaf color and pure aroma (Wang et al., 2016).

Recently, the processing techniques and technical equipment used in the Sichuan tea industry have improved substantially. However, the piling process used to develop the characteristic essence and flavor of dark tea is still based on traditional natural fermentation. Different successive microbial populations contribute to the process of natural fermentation of dark tea (Zhang et al., 2016b; Li et al., 2018b), and variations in environmental conditions can significantly alter the process that supports different populations in the microbial community. In short, the qualitative characteristics of dark tea, such as its essence and aroma that develop during the pile-fermentation process, are entirely dependent on the populations in the microbial community. Operational taxonomic units (OUTs) are variations among homologous sequence clusters of 16S rDNA of different microbial species with 97% identity threshold, which are employed to distinguish different microbial species present in samples (Schloss and Westcott, 2011). Principal component analysis (PCA) is a Euclidean based ordination technique that is used to express correlation in genomic sequencing data of microbes in the simplest way by removing noise and redundancy. While principal co-ordinate analysis (PCoA) is an independent algorithm non-constrained data dimensionality reduction method to express the correlation in genomic sequencing data of microorganisms (Minchin, 1987).

Yibin city in Sichuan province is situated on the bank of the Yangzi River, and dark tea cultivated here retains a special aroma due to the characteristic environmental conditions, unique pile-fermentation techniques used, and microbial resources. Although dark tea has been extensively studied for its growth, bioactive ingredients, and health benefits, little is known about the microbes involved in its qualitative improvement (Chen et al., 2013; Fu and Liu, 2015; Zhang et al., 2016a; Ge et al., 2019). To improve the unique qualities of dark tea, it is imperative to carefully analyze and effectively use these diverse microbial communities and their metabolic activities during pile-fermentation. The purpose of this study was to investigate the presence of different fungal species at different stages of pile-fermentation, so that beneficial fungal species can be artificially inoculated to improve aesthetic and nutritional value of dark tea. In this study, high-throughput sequencing was employed to investigate the composition of the diverse microbial communities present in Sichuan dark tea during pile-fermentation to improve processing.

## Materials and Methods

### Experimental Materials

Samples of Sichuan dark tea raw ingredients prepared from fresh leaves were obtained from the Sichuan Tea Industry Group Co., Ltd.^1^ No specific permission was required for sample collection for academic research. During fermentation, the leaves were mixed to ensure homogeneity; tap water was added as needed to maintain the solids content at 65-75% (w/v), and the temperature was maintained at 45-71°C. Samples were collected on days 0 (YC), 8 (W1), 16 (W2), 24 (W3), and 32 (W4) during fermentation. Three replicates were set up, and the collected samples were subjected to microbial analysis. The temperature at the center of the fermented tea pile, at a depth of 40 cm, was measured each day.

### DNA Extraction

For DNA extraction, 5 g of sample was weighed and immediately soaked in 25 ml of ddH_2_O. After stirring thoroughly, the sample was filtered through three layers of sterile gauze to remove large particles and then centrifuged at 12,298 × *g* for 10 min at 4°C. The supernatant was discarded, and the precipitate was used for genomic DNA (gDNA) extraction. We used the E.Z.N.A™ Fungal DNA Miniprep Kit (OMEGA, United States) according to the standard protocol to extract gDNA from the fungal tissues. The quality of the extracted gDNA was confirmed by running 2 μl of each sample on 1% agarose gel, which was visualized under a UV light in a gel documentation system (iBright imaging system, iBright 1500, ThermoFisher, United States; Wang et al., 2018a). The concentration of extracted DNA was recorded on spectrophotometer (Nanodrop 2000) at OD260/280, which was 2.1-42.5 ng/μl in each sample with triplicate manners.

### PCR Amplification and Sequencing Analysis

The primer pair ITS1F (5'-CTTGGTCATTTAGAGGAAGTAA-3') and ITS2R (5'-GCTGCGTTCTTCATCGATGC-3') was used to amplify the ITS1 region of the fungal ITS rDNA sequence (Li et al., 2017b) by PCR. Each reaction contained 4 μl of 5× FastPfu Buffer, 2 μl of 2.5 mmol/L dNTPs, 0.8 μl each of 5 μmol/L forward and reverse primer, 0.4 μl of FastPfu DNA Polymerase (TransStart®, AP221-01, China), 10 ng of DNA template, and ddH_2_O to 20 μl. The PCR conditions were as follows: an initial denaturation step at 95°C for 3 min, followed by 32 cycles of denaturation at 95°C for 30 s, annealing at 55°C for 45 s, and amplification at 72°C for 45 s, with a final amplification step at 72°C for 10 min, and storage at 10°C. To confirm amplification and amplicon size, 2 μl of each PCR product was separated by 2% agarose gel electrophoresis. The PCR product was used to construct a gDNA library, which was evaluated by high-fidelity Illumina MiSeq™ PE300 sequencing. Raw reads were trimmed, duplicate reads were merged according to PE read overlap, the quality of the reads was assessed, and splicing events were controlled. To distinguish samples, barcodes were assigned to each sample at the start and end of the sequencing based on the primer sequence. Finally, we obtained sufficient sequences, and their directions were corrected (Mukherjee et al., 2014).

### Sequencing and Phylogenetic Analysis

Phylogenetic clustering analysis was conducted using the Uparse OTU clustering software tool at a 97% identity threshold to identify representative sequences of operational taxonomic units (OTUs; Ye et al., 2017; Peng et al., 2019).^2^ The RDP classifier Bayesian algorithm was used to perform a taxonomic analysis of the OTUs at 97% identity threshold. The community composition and scientific classifications of each sample were established at the kingdom, phylum, class, order, family, genus, and species levels (Chen et al., 2016). We calculated the α-diversity of one sample of each using the Chao 1, ACE, Shannon, and Simpson indices to evaluate sequencing depth and coverage and to compare the abundance and diversity of the microbial communities in dark tea at different stages (Rogers et al., 2016). The number of common and unique OTUs in all samples was counted and a Venn diagram was constructed (Fouts et al., 2012). Variation in the composition of the OTUs in every sample was calculated using 97% identity threshold through PCA and principal coordinate analysis (PCoA; Calderón et al., 2017). The taxonomic analyses of all samples were compared at each classification level, and the R tool was used to construct community structure diagrams and histograms as combined analysis diagrams (Lu et al., 2016; Zhou et al., 2017).

### Statistical Analysis

All data were explained in mean values of standard deviation (SD) and analyzed by one-way analysis of variance (ANOVA). A Duncan multiple-comparison test was applied to detect variations among means of all the samples at *p* < 0.05 level of significance. All correlation and path coefficient analyses were performed with SPSS Statistics 20.0 (SPSS Inc., Chicago, IL, United States) and Excel 2019.

## Results

### Statistical Analysis of Sequencing Data

Illumina next generation DNA sequencing (NGS) of all five samples of Sichuan dark tea was performed in triplicate, and the results were analyzed to identify the identity threshold level among the fungal populations present in each sample using ITS metagenomics. The ITS1F/ITS2R universal primer pair was used to amplify the genomes of the different populations of fungi present in the microbial community of the dark tea (Supplementary Table S1). Incomplete and poor quality reads were eliminated. Ultimately, we obtained 334,593 high-quality fungal genomic sequences, with read lengths ranging from 249 to 278 bp (Supplementary Figure S1; Table 1).

**Table 1:** Characteristics of ITS sequences of the fungal populations in samples collected from Sichuan dark tea and the five time points during pile-fermentation.

Sample	Reads	Total bases	Average length (bp)
YC	69,242	16,428,484	276.6316
W1	65,769	16,436,511	249.9127
W2	71,262	19,845,494	278.4863
W3	65,242	17,154,692	262.9394
W4	63,078	15,839,408	277.6428

Column 1 is the sample name, column 2 is the number of sequencing reads in each sample, column 3 is the total number of bases, and column 4 is the average length, in base pairs, of each run.

**Figure 1:**
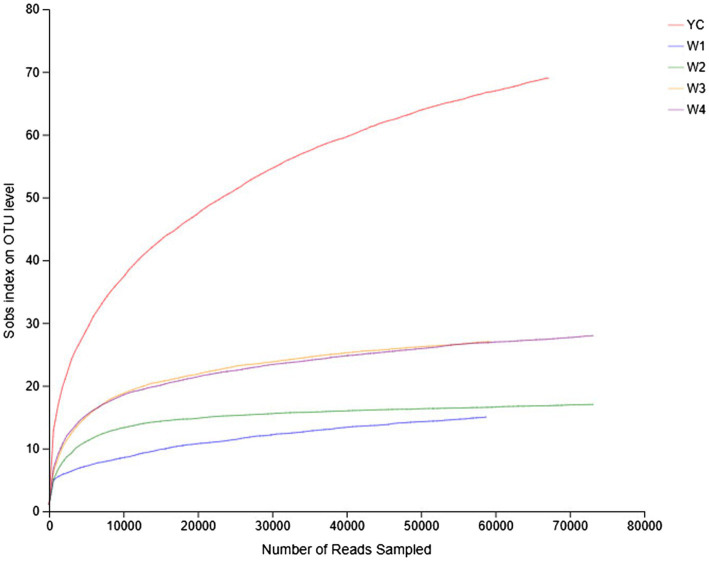
Rank abundance curves of all the samples. The abscissa represents the rank of the number of operational taxonomic units (OTUs) at a certain taxonomic level, and the ordinate represents the relative percentage of the number of species at that classification level. The position of the abscissa of the extension end point of the sample curve is the number of species in each sample. A smooth decline indicates higher species diversity in all the samples, while a rapid and steep decline indicates a high proportion of the major bacterial strains and low diversity.

Rarefaction curves reflect the richness and uniformity of the microbial species present in each sample of dark tea. The relative abundance curves of samples collected at W1 and W4 at 97% identity threshold appeared to have a small span and a steep decline (Figure 1). These results showed that the relative abundances of the OTUs between samples were remarkably different, the uniformity was very low, the fungal composition was relatively single, and diverse fungal species were present. On the other hand, the relative abundance curves of the YC and W2 samples were wider, and the curves were flat with a gradual decline, indicating that the species composition of the YC and W2 samples is richer, and the uniformity among species was higher (Figure 1). Except for the W2 samples, the microbial strains were not obvious and were relatively concentrated.

### Operational Taxonomic Unit Cluster Analysis

**Figure 2:**
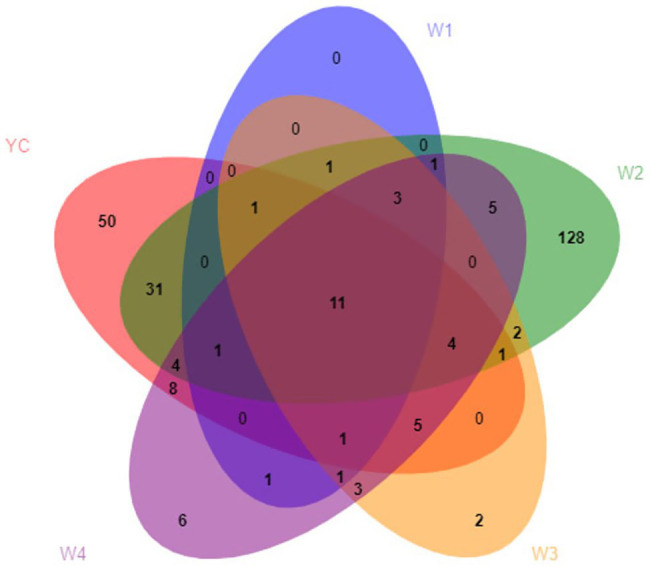
Venn analysis of the OTUs. Different groups are shown in different colors, and the numbers in the overlapping portions represent the number of species common to all the groups.

During the OTU analysis, all non-repetitive single sequences were separated from the optimized sequences, and redundant sequences were eliminated. Except for single sequences, OTU clustering analysis of non-repetitive sequences was performed at 97% identity threshold. Subsequently, all the chimeric sequences were removed, and only a single representative sequence for each OTU was included in the analysis. Clustering analysis of valid data yielded 270 fungal OTUs, which were classified into seven phyla, 22 classes, 41 orders, 85 families, 128 genera, and 184 species (Supplementary Table S2). Only 11 fungal OTUs were common among all five groups, 14 OTUs were common between W1 and YC, 53 OTUs were common between W2 and YC, 23 OTUs were common between W3 and YC, and 34 OTUs were common between W4 and YC (Figure 2).

### Microbial Diversity Analysis

The coverage threshold for the sequences from the dark tea samples collected at all five time points was >99%, indicating that it represents the true fungal populations in the microbial community of each sample. To estimate the species richness or total number of microbial species in each sample, the sequencing results were analyzed using ACE and Chao 1 indices (Chernov et al., 2015; Wang et al., 2017). The samples with the highest abundance levels, according to the ACE and Chao1 values (at 89.27 and 84.57%, respectively), were observed in the YC group (Table 2). In contrast, the samples with the lowest abundance levels, according to the ACE and Chao1 values (at 28.16 and 20.00%, respectively), were observed in group W1. The remaining groups had intermediate abundance levels according to the ACE and Chao 1 values, which were 45.03 and 44.43 in W2, 33.40 and 36.00 in W3, and 49.53 and 46.27 in W4, respectively. The overall microbial species abundance in the Sichuan dark tea samples collected at the tested time points was in descending order: YC > W4 > W2 > W3 > W1 (Table 2).

**Table 2:** Indices of fungal community richness and diversity of Sichuan dark tea.

Sample	Shannon	Simpson	ACE	Chao1	Coverage
W1	0.84	0.49	28.16	20.00	0.999915
W2	0.77	0.55	45.03	44.43	0.999928
W3	0.60	0.70	33.40	36.00	0.999927
W4	0.94	0.49	49.53	46.27	0.999825
YC	1.09	0.46	89.27	84.57	0.999650

The first column is the sample names, and columns 2-4 are the Shannon, Simpson, ACE, and Chao 1 diversity index values of each sample. The last column is average coverage diversity index values.

The Shannon and Simpson indices are comprehensive indicators that reflect the proportion of different species in a diverse community (Chernov et al., 2015; Wang et al., 2017). The Shannon-Weiner index is directly proportional to the richness and diversity of different populations in a community and is negatively correlated with the Simpson index. An Elevated Shannon-Weiner index value indicates higher species diversity in a microbial community, whereas an elevated Simpson index value indicates low species diversity. The Shannon-Weiner values of the samples were, in descending order: YC >W4 > W1 > W2 > W3. Similarly, the Simpson values of the samples were in descending order: W3 > W2 > W1 > W4 > YC. The highest Shannon-Weiner index value and lowest Simpson index value of the YC samples showed both high fungal species richness and diversity. With continued piling time, the fungal diversity first decreased and then exponentially increased in YC samples, and the highest diversity was observed before piling.

### Fungal Community Structure Analysis

**Figure 3:**
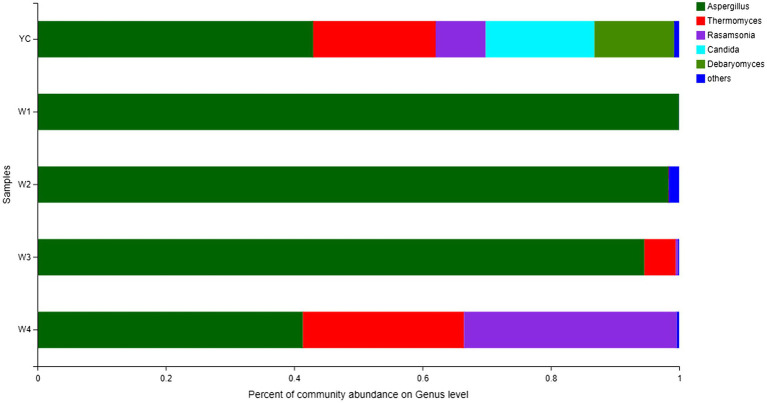
Fungal community structure bar plot at the genus level. Each ordinate is the sample name, and the abscissa is the proportion of each species in a sample. Different species are shown as columns of different colors, and the size and proportion of each species is represented by the length of the column.

**Figure 4:**
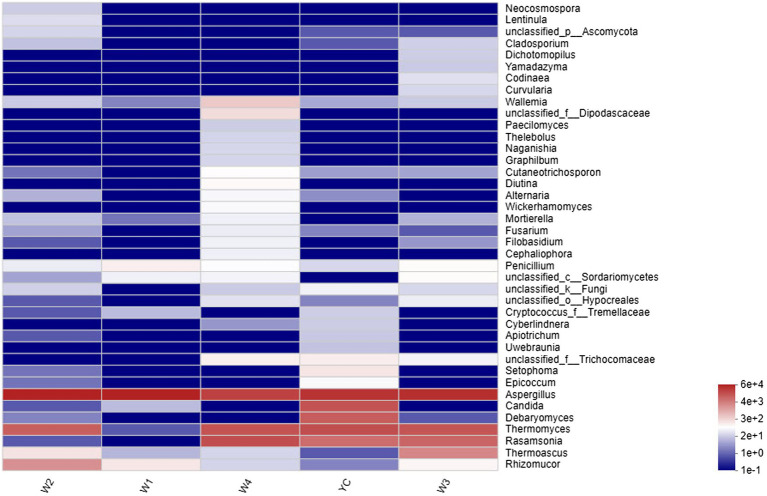
Community heatmap at the genus level. The abscissa represents the group name, and the ordinate represents the species name. The changes in the abundance of different species in each sample are displayed as a color gradient in a color block. The bar on the right side of the figure shows the abundance as a color gradient.

Comprehensive qualitative or quantitative compositional analysis of the 40 most frequent populations in the microbial communities of Sichuan dark tea at the genus level were used to construct a histogram (Figure 3) and heatmap (Figure 4). The analysis showed that *Aspergillus* was the most abundant in all the tested samples. The abundance of *Aspergillus* increased exponentially to 99.3% at W1 and then decreased over time. In addition to *Aspergillus*, four other genera, *Thermomyces*, *Candida*, *Debaryomyces*, and *Rasamsonia*, also displayed higher abundances in the YC group, at 19.1, 16.9, 12.4, and 7.8%, respectively. In the W2 group, the genus with the highest abundance was also *Aspergillus*, although *Rhizomucor* (13.4%) and *Thermomyces* (3.1%) were also frequent (Figure 3). The possible reason of higher fungal species during early stages could be the presence of fungal spores in raw material used in pile-fermentation.

Other highly abundant fungal genera in W3 were *Thermomyces*, *Rasamsonia*, and *Thermoascus*, while the highly abundant fungal genera in group W4 were *Rasamsonia* and *Thermomyces* (Figure 3). The highest abundance of *Aspergillus* species was obvious in the heat map (Figure 4). Notably, although information about unclassified *Trichocomaceae* and *Ascomycota* could not clearly be distinguished at the genus level, these likely are mostly *Aspergillus cristatum*. In addition, *Candida* and *Debaryomyces* were also detected in the dark tea samples after pile-fermentation, but their abundance was less than 1% (Figure 4).

### Fungal Community Structure

**Figure 5:**
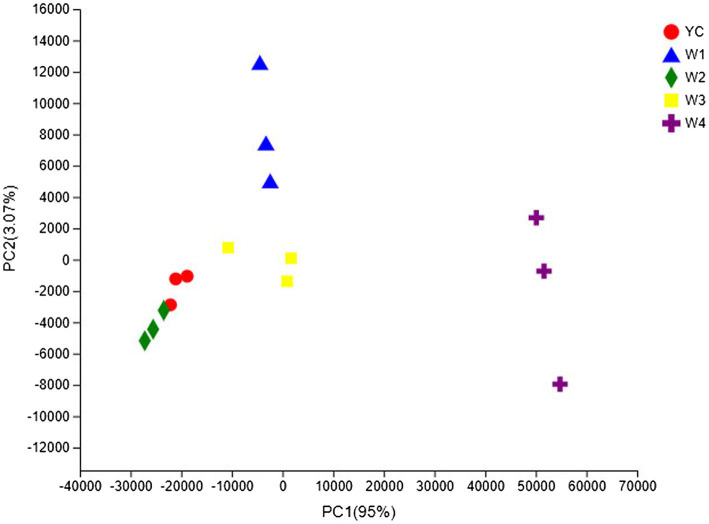
Multiple sample principal component analysis (PCA) of the OTUs. Both selected principal component axes are represented by the *x*- and *y*-axes, and the percentage represents the difference in sample composition as determined by the principal component; the scales of the *x*- and *y*-axes represent the relative distances. Samples are shown as different color points or shapes in different groups. The closeness of two points or shapes represents the similarity between the species composition of two samples.

**Figure 6:**
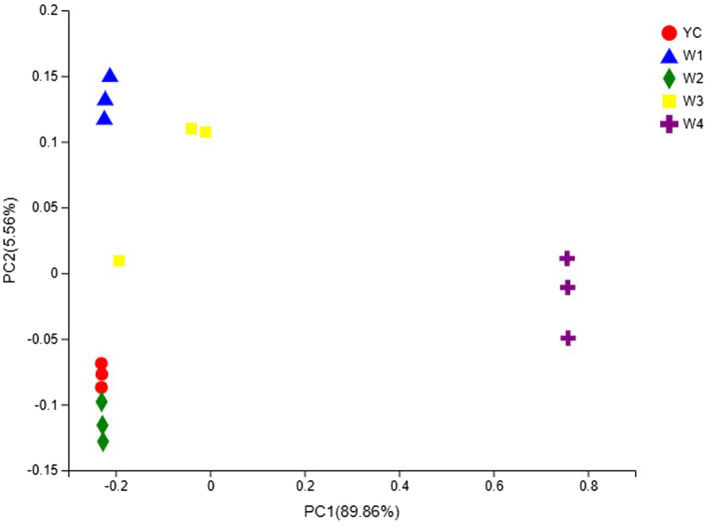
Multiple sample principal coordinate analysis (PCoA) of OTUs. Both selected principal component axes are represented by the *x*- and *y*-axes, and the percentage represents the difference in sample composition as determined by the principal component; the scales of the *x*- and *y*-axes are the relative distances. Samples are shown as different color points or shapes in different groups. The closeness of two points or shapes represents the similarity between the species composition of two samples.

The OTU composition of all Sichuan dark tea samples was analyzed *via* PCA and PCoA to undermine the Euclidean distances and dissimilarity index. The highly abundant fungal genera in all the samples were used to generate PCA and PCoA maps (Figures 5, 6). PC1 and PC2 were drawn along the *x*- and *y*-axes, respectively, and their combined total contribution to the PCA score was 98.07% (Figure 5) and that to the PCoA score was 95.42% (Figure 6). Notably, YC and W2 were closely clustered but distant from the rest of the samples collected at W1, W3, and W4. In contrast, the YC and W4 samples disintegrated along PC1 and PC2. These results showed obvious differences in the fungal communities of Sichuan dark tea at the different stages of pile-fermentation.

The fungal communities in the dark tea samples collected at pile-fermentation stages YC, W1, W2, and W3 were remarkably similar, but quite different from those in sample collected at W4. The community at W4 was separate from the communities in the other samples. These results led us to conclude that the late stage of pile-fermentation (a critical stage of dark tea production; W4) contained prominent factors that affected the population structure in the fungal community of Sichuan dark tea. Note that the total number of samples and the unique results of individual samples have implications for the results of the entire analysis, which could be minimized by increasing the total number of samples.

### Functional Classification of the Fungi in Sichuan Dark Tea

**Figure 7:**
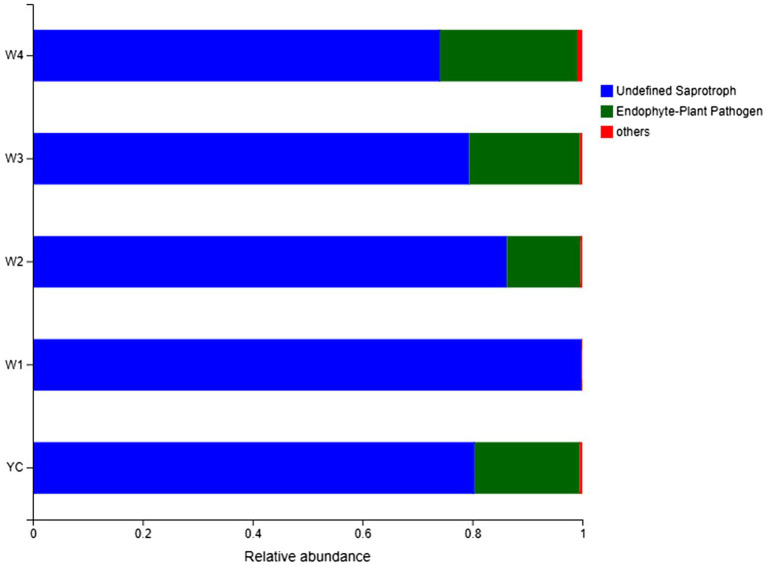
FUNGuild analysis of fungal functional groups. The relative abundance of the Guild in different groups or samples is on the *x*-axis, and groups or samples are on the *y*-axis. According to the variation in functional groups, FUNGuild can calculate the abundance of each fungal species and their functional classification in each sample.

Guild/ecological guild refer to the relationship between closely or distantly related species inhabiting similar or different environments in similar ways (Schmidt et al., 2019). FUNGuild (Nguyen et al., 2016) is a sequencing and analysis platform-independent Python-based tool that was employed for functional classification of the fungal OTUs in each sample of Sichuan dark tea.^3^ The relative abundance of two ecological guilds, undefined saprotrophs and endophytes/plant pathogens, inhabiting the Sichuan dark tea samples collected at 8-day intervals during piling obtained by FUNGuild was more than 99% (Figure 7). The population abundance information for each OTU was also obtained, which is a prerequisite for understanding the sources and pathways of different microbes. These results showed that a considerable portion of the fungal species was assimilated with the harvested raw materials for the piling of Sichuan dark tea. Herein, the detection of a higher number of endophytes/plant pathogens is an indicator of the existence of a wide range of fungal communities present in the raw material for dark tea (Figure 7).

## Discussion

The length of the piling cycle required to develop the unique flavor of Sichuan dark tea depends predominantly on the composition of the microbial community in the pile. A variety of microorganisms are involved in the pile-fermentation of Sichuan dark tea. In the past, due to technical limitations, determining the composition of the microbial community responsible for the essence/flavor development of dark tea required traditional separation and culture-dependent methods, which are time-consuming, laborious, costly, and ultimately non-robust, because of the failure to cultivate several microbes. Therefore, only fungi which can be cultured were explored, and it was impossible to fully elucidate the unique role of microbes during pile-fermentation (Li et al., 2017a). In this study, we used a robust high-throughput sequencing method and obtained 334,593 high-quality fungal sequences, with an average length of 269.12 bp, with an aim to determine the composition of the fungal community during pile-fermentation of dark tea (Table 1). All the sequences were classified into OTUs at 97% identity threshold, and 270 fungal OTUs were obtained by clustering valid data, which were divided into seven phyla, 22 classes, 41 orders, 85 families, 128 genera, and 184 species.

Based on diversity indices, the abundance of the fungal community changed during pile-fermentation, which first decreased and then increased. The highest fungal diversity was observed at the end of pile-fermentation, which was higher than that in the raw materials, indicating that the highest number of fungal species was present at the end of fermentation. A possible reason for this could be a higher rate of bacterial growth over fungal growth at the initial stage of pile-fermentation due to temperature conditions that were more suitable for bacteria. Bacterial growth appeared to be reduced at the middle and final stages due to the increase in temperature. Investigations on the possible role of bacteria during pile-fermentation are underway. Notably, at the final stages of pile-fermentation, a series of catabolic reactions, such as anaerobic bacterial respiration and decay of the vegetative parts of tea plants, provided a suitable temperature and essential nutrients to support the growth and reproduction of fungi. A gradual increase in piling time resulted in an increase in the diversity index of the fungal populations, indicating that piling time and fungal diversity are directly proportional; a similar result was obtained in a previous study (Li et al., 2018a).

The similarity of the fungal community structure was lower in the YC samples and then gradually increased in samples collected at stages W1-W3, which is consistent with previous findings (Zhang et al., 2016a), regarding the structural variations in the microbial community during the fermentation of Puer tea (Chen et al., 2013). The abundance of the fungal community increased exponentially in all W4 samples when compared to that of the W1, W2, and W3 samples, indicating that the W4 step of pile-fermentation is a key time point for flavor and quality development. The fungal population was relatively rich at the YC time point, indicating that the raw materials used in the pile-fermentation of dark tea is a secondary factor affecting the variation in fungal community structure (Zhao et al., 2017).

During pile-fermentation of dark tea, the growth and reproduction of microbes are interdependent, and a balanced and stable mechanism will eventually develop to generate the conditions necessary for post-fermentation processing. This study revealed that the most abundant fungal genera involved in the pile-fermentation of Sichuan dark tea are *Aspergillus*, *Thermomyces*, *Candida*, *Debaryomyces*, *Rasamsonia*, *Rhizomucor*, and *Thermoascus* (Figures 3, 4). Given their abundance, the abovementioned abundant fungal species likely play pivotal roles in development of the unique aroma and active ingredients of dark tea. Numerous *Aspergillus* species, such as *A. cristatum* (*Eurotium cristatum*) and *A. carbonarius*, are economically important and are widely employed in food biotechnology to enhance the nutritional value of food products, such as tea and coffee, due to the various metabolites they produce during fermentation (Samson et al., 2014; De Souza et al., 2021). Many *Aspergillus* species produce and secrete various enzymes, such as α-amylase, glucoamylase, cellulase, pectinase, xylanase, hemicellulase, and protease, which are applied on an industrial scale to improve the taste of food items by breaking down proteins or lipids and developing unique flavors (Ward et al., 2005). Additionally, *A. cristatum* is widely used in making crimson soup from dark tea, and lovastatin, a chemical secreted by this genus, is a statin that lowers cholesterol levels (Shi et al., 2005; Zhao et al., 2013). Further studies are needed to explore the probiotic properties of individual fungal species and their role in nutrition enhancement.

Some species of *Candida*, such as *Candida etchellsii*, *C. milleri*, *C. rugosa*, and *C. tropicalis*, can grow on liquor waste and are used in the food and feed industries to improve nutritional value and taste and as cell factories for the production of single-cell proteins (Bourdichon et al., 2012). *Debaryomyces hansenii* (anamorph *C. famata*) is also employed in the food industry for surface ripening of cheese and meat products, the production of riboflavin (vitamin B2), bioconversion of xylose into xylitol sweetener, and the biosynthesis of arabinitol and pyruvic acid (Breuer, 2010). During dark tea pile-fermentation, some fungi, such as *Rhizomucor*, can secrete antibacterial substances that inhibit the growth of bacteria, which makes the tea safer to drink. *Rhizomucor* is also involved in the degradation of bio-waste and carbon uptake for the biosynthesis of various useful enzymes, such as 1,4-β-xylosidase, endo 1,4-β-glucanase, phosphatase, chymosin, protease, and alcohol dehydrogenase, which are important for the flavor development of dark tea (Zhang et al., 2013). *Rasamsonia* produces cellulase, hemicellulase, pectinase, and starch-degrading enzymes, and catalyzes various reactions, such as oxidation, oxido-reduction, and proteolysis, which are important for dark tea fermentation (Waters et al., 2010). The *unclassified_Trichocomaceae* and *unclassified_Ascomycota* reads identified in this study are probably from *A. cristatum*, commonly known as “golden flower,” which has a major impact on the pile-fermentation process of dark tea (Ge et al., 2016; Li et al., 2017a). Several indole alkaloids and indole diketopiperazine alkaloids can be extracted from a culture of *A. cristatum*, which possess brine shrimp killing activity, antibacterial activity against *E. coli*, radical scavenging activity against DPPH radicals, and marginal attenuation of 3T3L1 pre-adipocytes (Du et al., 2017). Therefore, an artificial inoculation of dark tea with these fungi could improve its health benefits and could be used to produce probiotic dark tea.

It is of immense interest to determine the functional classification, abundance, source, and metabolic pathways of the microbes involved in the flavor development of dark tea. The source of the external fungi was the spores present on the raw material, and their relative abundance was high in each pile-fermentation. The high number of endophytes shows the broad range and diversity of the fungi in the raw materials of dark tea (Crous et al., 2006). Our findings provide deep insights in fungal community structure, which can be applied in piling stage-specific fungal inoculation that may improve the nutritional quality and aesthetic value of dark tea (Bressani et al., 2021) to overcome the challenge of malnutrition, which is a key point for one of the “Sustainable Development Goals (SDGs)” of UNO, to “Establish Good Health and Well-Being.”

## Conclusion

The Sichuan dark tea is being processed *via* pile-fermentation to improve its nutritional value and aroma. It is believed that microorganisms, such as fungi and bacteria, present during each piling cycle play crucial role in the development of nutritional value and aroma during fermentation, but little is known about their composition. A deep insight in composition of these microbes will definitely pave way to artificially and pile specific inoculation of symbionts to improve the nutritional and aesthetic values of dark tea. Robust next generation sequencing analysis revealed that following fungal genera; *Aspergillus*, *Thermomyces*, *Candida*, *Debaryomyces*, *Rasamsonia*, *Rhizomucor*, and *Thermoascus* were highly abundant during piling process, of which *Aspergillus* was the most abundant. Notably, the highest number of fungal species were observed before piling stage (YC), which were gradually decreased in subsequent piling stages (W1 and W2), and then gradually increased again at piling stages W3 and W4. Among all, the highly abundant fungal genus was *Aspergillus* present at W4 piling stage, which is of medical and commercial importance. In conclusion, W3 and W4 are suitable stages for inoculation of symbiotic fungal species to develop essence and nutritional value of Sichuan dark tea.

## Data Availability Statement

The datasets presented in this study can be found in online repositories. The names of the repository/repositories and accession number(s) can be found at: European Nucleotide Archive (ENA), Accession number PRJEB46392. Our study unique name is ena-STUDY-Manzar Abbas-16-07-2021-16:22:51:147-260.

## Author Contributions

KY and MA designed the experiments. KY, MA, LM, ZP, QL, XZ, and HC performed the experiments. KY, MA, AE-S, LY, and XZ analyzed the data. MA and KY wrote the manuscript. All authors contributed to the article and approved the submitted version.

## Conflict of Interest

HC, ZP, and LY were employed by the company Sichuan Province Tea Industry Group Co., Ltd.

The remaining authors declare that the research was conducted in the absence of any commercial or financial relationships that could be construed as a potential conflict of interest.

## Publisher’s Note

All claims expressed in this article are solely those of the authors and do not necessarily represent those of their affiliated organizations, or those of the publisher, the editors and the reviewers. Any product that may be evaluated in this article, or claim that may be made by its manufacturer, is not guaranteed or endorsed by the publisher.

## Footnotes

^1^ http://www.scteag.com/

^2^ http://drive5.com/uparse/

^3^ http://www.funguild.org
