# Supplementary material for: Analysis of the Fungal Diversity and Community Structure in Sichuan Dark Tea During Pile-Fermentation
Source: Front Microbiol. 2021 Aug 5;12:706714. doi: 10.3389/fmicb.2021.706714 (PMC8375752; doi:10.3389/fmicb.2021.706714)
Supplement: Supplementary file 1 [file Data_Sheet_1.zip › Table S1.docx]

Supplemental Table S1. ITS sequencing analysis of all samples of Sichuan dark tea.

| Sample | Valid reads | Average length | Total bases | Q30 | Q20 | Primer |
| --- | --- | --- | --- | --- | --- | --- |
| FS1_1 | 48496 | 256.1636 | 12422912 | 97.22891 | 98.67409 | ITS1F_ITS2R |
| FS1_2 | 48104 | 259.0211 | 12459953 | 97.11472 | 98.60617 | ITS1F_ITS2R |
| FS1_3 | 59123 | 232.4379 | 13742428 | 98.59548 | 99.35798 | ITS1F_ITS2R |
| FS2_1 | 65941 | 258.7213 | 17060338 | 97.93803 | 98.90915 | ITS1F_ITS2R |
| FS2_2 | 69236 | 247.7674 | 17154421 | 98.465871 | 99.16726 | ITS1F_ITS2R |
| FS2_3 | 72817 | 260.4405 | 18964494 | 97.166647 | 98.62198 | ITS1F_ITS2R |
| FS3_1 | 56092 | 264.6035 | 14842141 | 97.08821 | 98.63747 | ITS1F_ITS2R |
| FS3_2 | 72155 | 260.8012 | 18818111 | 97.125365 | 98.62515 | ITS1F_ITS2R |
| FS3_3 | 59370 | 260.7316 | 15479635 | 96.955632 | 98.57349 | ITS1F_ITS2R |
| FS4_1 | 53142 | 277.4004 | 14741614 | 96.567391 | 98.40062 | ITS1F_ITS2R |
| FS4_2 | 56961 | 280.5061 | 15977910 | 96.61952 | 98.43624 | ITS1F_ITS2R |
| FS4_3 | 74492 | 261.81 | 19502752 | 97.111869 | 98.61797 | ITS1F_ITS2R |
| Y_1 | 143619 | 276.6316 | 39729550 | 96.97181 | 98.60665 | ITS1F_ITS2R |
| Y_2 | 69242 | 237.2618 | 16428484 | 98.55723 | 99.3633 | ITS1F_ITS2R |
| Y_3 | 47544 | 270.5493 | 12862997 | 98.45662 | 99.30744 | ITS1F_ITS2R |

Note: First column is the sample name, and the 2-7 columns are sample-related information, followed by Valid reads, Average length, Total bases, Q30, Q20 and Primer.
