# Supplementary figures and images for: Analysis of the Fungal Diversity and Community Structure in Sichuan Dark Tea During Pile-Fermentation
Source: Front Microbiol. 2021 Aug 5;12:706714. doi: 10.3389/fmicb.2021.706714 (PMC8375752; doi:10.3389/fmicb.2021.706714)

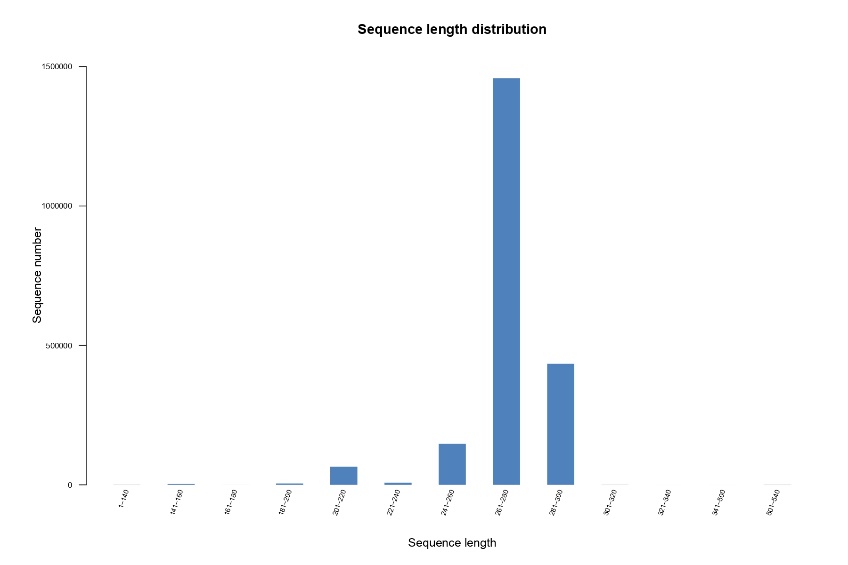


**Supplemental Figure S1.** The x-axis is the length interval, and the y-axis is the number of sequences.

Supplement: Supplementary file 1 [file Data_Sheet_1.zip › Figure S1.docx]
